# IFI16 Impacts Metabolic Reprogramming during Human Cytomegalovirus Infection

**DOI:** 10.1128/mbio.00435-22

**Published:** 2022-04-14

**Authors:** Gloria Griffante, Weronika Hewelt-Belka, Camilla Albano, Francesca Gugliesi, Selina Pasquero, Sergio Fernando Castillo Pacheco, Greta Bajetto, Paolo Ettore Porporato, Erica Mina, Marta Vallino, Christian Krapp, Martin Roelsgaard Jakobsen, John Purdy, Jens von Einem, Santo Landolfo, Valentina Dell’Oste, Matteo Biolatti

**Affiliations:** a Department of Public Health and Pediatric Sciences, University of Turin, Turin, Italy; b Department of Analytical Chemistry, Faculty of Chemistry, Gdańsk University of Technology, Gdańsk, Poland; c Department of Molecular Biotechnology and Health Sciences, University of Turin, Turin, Italy; d Institute for Sustainable Plant Protection, CNR, Turin, Italy; e Department of Biomedicine, Aarhus University, Aarhus, Denmark; f Department of Immunobiology, University of Arizona, Tucson, Arizona, USA; g Institute of Virology, Ulm University Medical Center, Ulm, Germany; Columbia University College of Physicians & Surgeons

**Keywords:** IFI16, glucose and lipid metabolism, human cytomegalovirus, lipidomics, virus-host interactions

## Abstract

Cellular lipid metabolism plays a pivotal role in human cytomegalovirus (HCMV) infection, as increased lipogenesis in HCMV-infected cells favors the envelopment of newly synthesized viral particles. As all cells are equipped with restriction factors (RFs) able to exert a protective effect against invading pathogens, we asked whether a similar defense mechanism would also be in place to preserve the metabolic compartment from HCMV infection. Here, we show that gamma interferon (IFN-γ)-inducible protein 16 (IFI16), an RF able to block HCMV DNA synthesis, can also counteract HCMV-mediated metabolic reprogramming in infected primary human foreskin fibroblasts (HFFs), thereby limiting virion infectivity. Specifically, we find that IFI16 downregulates the transcriptional activation of the glucose transporter 4 (GLUT4) through cooperation with the carbohydrate-response element-binding protein (ChREBP), thereby reducing HCMV-induced transcription of lipogenic enzymes. The resulting decrease in glucose uptake and consumption leads to diminished lipid synthesis, which ultimately curbs the *de novo* formation of enveloped viral particles in infected HFFs. Consistently, untargeted lipidomic analysis shows enhanced cholesteryl ester levels in IFI16 KO versus wild-type (WT) HFFs. Overall, our data unveil a new role of IFI16 in the regulation of glucose and lipid metabolism upon HCMV replication and uncover new potential targets for the development of novel antiviral therapies.

## INTRODUCTION

Human cytomegalovirus (HCMV) is a betaherpesvirus with a worldwide seroprevalence of up to 90% (1). It is often asymptomatic in immunocompetent adults, but it can cause severe and sometimes fatal diseases in immunocompromised individuals and neonates (2–5).

Due to its ability to establish a lifelong latent or chronic/permanent infection, HCMV may also lead to other unexpected long-term health sequelae. For example, HCMV infection was first associated with atherosclerosis in 1987 (6), a connection further supported by a number of studies showing HCMV infection of endothelial cells (ECs) to be a crucial event for atherosclerosis development. Indeed, once aortic ECs are infected with HCMV, they become a source of viral spread, further infecting the vascular tissue, altering cellular processes, and eventually leading to atherosclerosis (7).

The aforementioned events are not surprising in light of numerous strategies devised by HCMV to exploit cellular pathways for its own benefit (8, 9). One such strategy consists of inducing metabolic reprogramming of host cells to improve its replication and release (10–14). An overall increase in metabolites from glycolysis, tricarboxylic acid (TCA) cycle, and pyrimidine synthesis pathways was in fact observed following HCMV-mediated upregulation of enzymes involved in these pathways (15). In particular, HCMV-infected fibroblasts displayed enhanced glucose consumption and lactate production, while glucose withdrawal impaired virus replication in these cells (16–19).

Glucose transporter 4 (GLUT4) has long been known to be one of the main targets of HCMV activity (20). In particular, increased GLUT4 levels were found in infected fibroblasts, whereas GLUT1, whose glucose transport capacity is lower than that of GLUT4, was inhibited through a mechanism involving the HCMV major immediate early protein IE72 (20). HCMV-mediated GLUT4 upregulation relies on carbohydrate response element binding protein (ChREBP) (21) and AMP-activated protein kinase (AMPK) (22, 23), both of which are induced during infection. Conversely, inhibition of ChREBP or AMPK signaling counteracts the metabolic changes induced by HCMV, thus curbing viral replication (21–23). Finally, GLUT4 translocation to the cell surface has been observed in infected cells, accompanied by an increase in cytoplasmic glucose for *de novo* fatty acid biosynthesis (20), which will then be available for viral envelopment (24).

It is widely established that HCMV can persist in the host thanks to its multilayered ability to escape from immune surveillance. One of the key players in this process is gamma interferon (IFN-γ)-inducible protein 16 (IFI16), which we have previously shown to act as a restriction factor (RF) for HCMV replication through transcriptional downregulation of the DNA polymerase UL54 (25). However, HCMV has evolved several strategies to escape from the restriction activity of IFI16, one of which relies on IFI16 nuclear delocalization, followed by incorporation into newly formed virions and expulsion from the cell (26, 27). Thus, we asked whether IFI16 could also act as an RF against HCMV within the metabolic compartment, which is essential to achieve a productive HCMV infection (12).

Here, we provide the first evidence of IFI16 acting as an inhibitor of lipid synthesis in HCMV-infected primary human foreskin fibroblasts (HFFs), whose ultimate effect is the release of virus particles with reduced infectivity. Overall, our findings reveal an unprecedented function of IFI16 based on its ability to keep HCMV replication in check by regulating HCMV-induced metabolic reprogramming.

## RESULTS

### IFI16 hampers glucose uptake upon HCMV infection.

**FIG 1:**
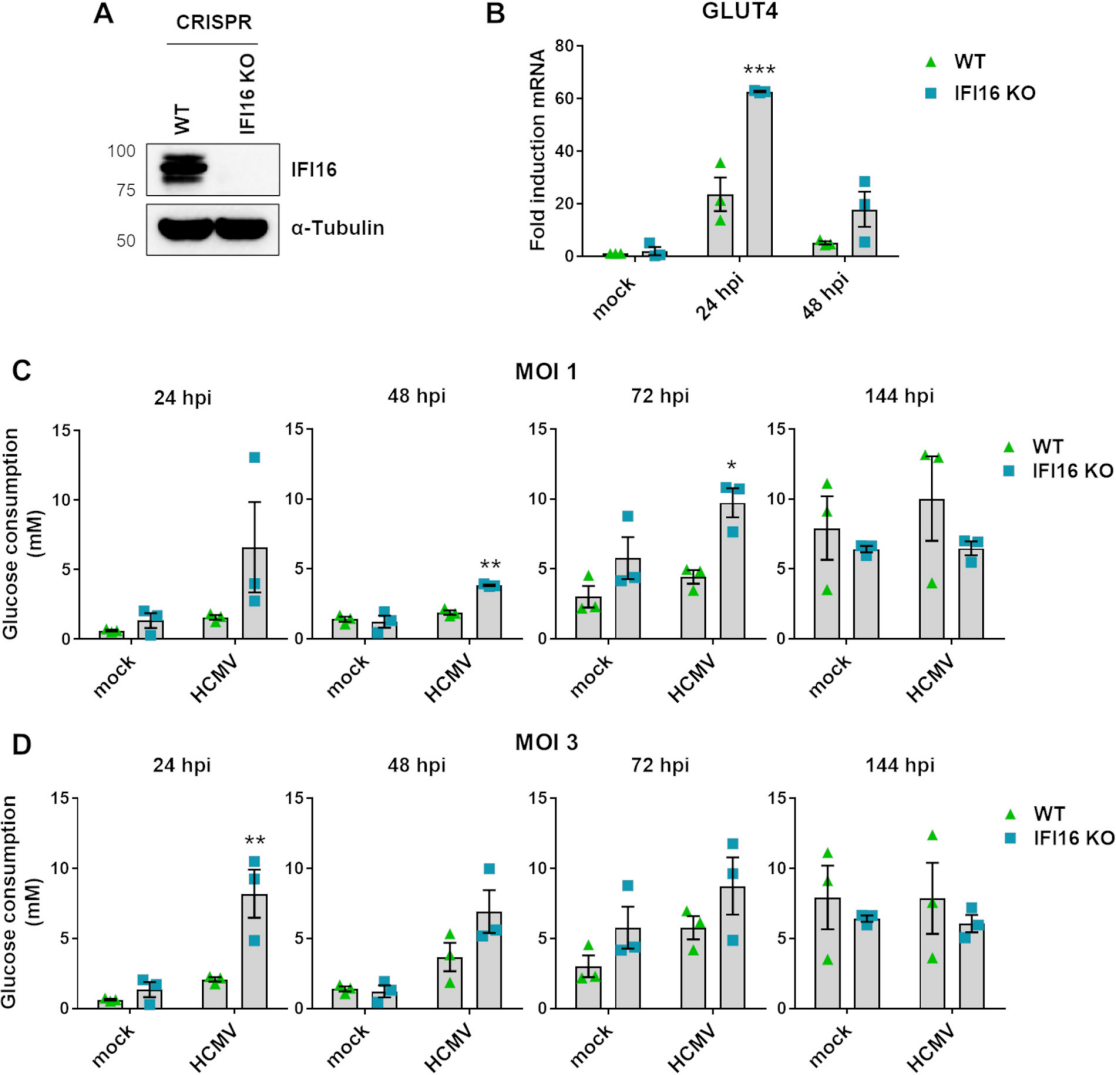
IFI16 involvement in glucose consumption. (A) A knockout gene variant of IFI16 (IFI16 KO) was generated from HFFs using CRISPR/Cas9 technology. Wild-type HFFs (WT) were used as controls. The efficiency of IFI16 protein depletion was assayed by Western blotting using a monoclonal antibody against human IFI16 or α-tubulin as a loading control. (B) WT and IFI16 KO HFFs were infected with HCMV (MOI, 1). To measure GLUT4 expression levels, total RNA was isolated at 24 and 48 hpi and subjected to RT-qPCR. Values were normalized to the housekeeping GAPDH gene mRNA and plotted as fold induction relative to mock-infected WT HFFs (set at 1). Bars show means and SEM from three independent experiments (***, *P < *0.001; two-way analysis of variance [ANOVA] followed by Bonferroni’s posttests, for comparison of WT versus IFI16 KO cells). (C and D) Culture medium was collected from mock- or HCMV-infected IFI16 KO or WT HFFs (MOI of 1 [C] and 3 [D]) at 24, 48, 72, or 144 hpi. Glucose concentration in the supernatants was measured as described in Materials and Methods. Bars show means and SEM from two independent experiments (*, *P < *0.05; **, *P < *0.01; two-way ANOVA followed by Bonferroni’s posttests, for comparison of WT versus IFI16 KO HFFs).

As IFI16 mediates intrinsic resistance to HCMV (25, 28), we asked whether it might counteract the metabolic rewiring promoted by HCMV infection. To this end, we generated gene knockout (KO) variants of HFFs through clustered regularly interspaced short palindromic repeat (CRISPR)/Cas9 technology. Primary cell lines carrying mutations in genes encoding IFI16 (IFI16 KO) were generated based on two different guide RNAs (gRNAs). Targeting effectiveness was verified by Western blotting (Fig. 1A) and tracking of indels by decomposition (TIDE) analysis in comparison with the parental cell line (WT), as we reported previously (27).

Since HCMV exploits GLUT4 to carry hexose sugars across the membrane (20), we first sought to determine whether IFI16 expression would affect GLUT4 expression and GLUT4-mediated glucose uptake in HCMV-infected HFFs. As shown in Fig. 1B, HCMV (laboratory strain AD169) infection of WT HFFs triggered lower levels of GLUT4 mRNA expression compared to those observed in IFI16 KO cells at 24 and 48 h postinfection (hpi). Particularly, the levels of GLUT4 transcript at 24 hpi in IFI16 KO HFFs were significantly higher (2.6-fold) than those observed in WT HFFs. Thus, IFI16 expression is required to tackle HCMV-mediated GLUT4 mRNA upregulation in the early stages of viral infection.

Given that enhanced GLUT4 expression results in increased glucose uptake (20), we sought to assess glucose consumption in IFI16 KO versus WT HFFs following HCMV infection. For this purpose, we measured glucose concentrations in supernatants from WT and IFI16 KO HFFs (mock or HCMV infected) following infection kinetics from 24 to 144 hpi, using multiplicities of infection (MOI) of 1 and 3 (Fig. 1C and D, respectively). As shown in Fig. 1C and D, glucose consumption was much faster in the cell culture medium of HCMV-infected IFI16 KO HFFs than in that of HCMV-infected WT cells in an MOI-dependent manner. Particularly, a significant enhancement of glucose consumption in IFI16 KO cells was observed at 48 and 72 hpi at an MOI of 1, followed by later pairing (Fig. 1C). A similar and earlier trend of glucose utilization was also recorded at 24 hpi in IFI16 KO cells incubated with HCMV at an MOI of 3 (Fig. 1D).

Taken together, these results confirm that IFI16 acts as a transcriptional repressor of GLUT4, thereby reducing glucose consumption in HCMV-infected cells during the early stages of viral infection.

### ChREBP is induced in the absence of IFI16.

**FIG 2:**
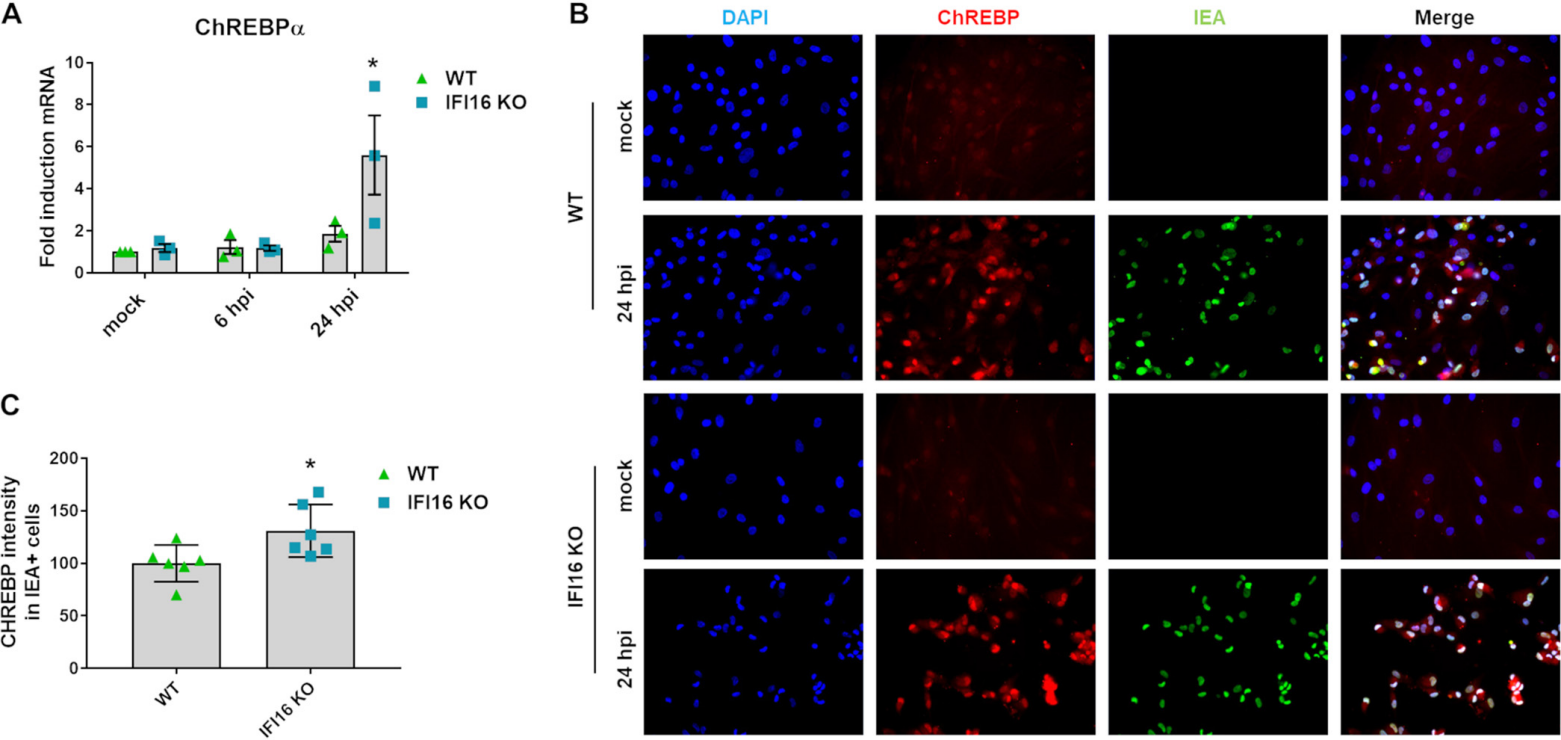
ChREBP modulation in HCMV WT- and IFI16 KO-infected cells. WT and IFI16 KO HFFs were infected with HCMV (MOI, 1), for the indicated times. (A) Total RNA was isolated, and ChREBPα mRNA levels were measured by RT-qPCR, normalized to GAPDH mRNA, and plotted as fold induction relative to mock-infected WT cells (set at 1). Bars show means and SEM from three independent experiments (*, *P < *0.05; two-way ANOVA followed by Bonferroni’s posttests, for comparison of WT versus IFI16 KO cells). (B) Representative immunofluorescence (IF) images of HCMV-infected HFFs in the presence of 10% HCMV-negative human serum. The ChREBP protein is stained in red, whereas the HCMV protein immediate early antigen (IEA) is stained in green. Cell nuclei are visualized by DAPI (blue). (C) Amount of ChREBP localized in the nucleus of HCMV IEA-positive IFI16 KO cells compared to WT cells calculated from 2 different fields for each experiment. IF staining intensity is represented as fold induction relative to WT-infected cells (set at 100%). Bars show means and SEM from three independent experiments (*, *P < *0.05; unpaired *t* test).

As Yu et al. (20) showed previously that ChREBP is a critical regulator of HCMV-induced metabolic alterations by acting directly on GLUT4 and GLUT2 expression, we assessed the ability of IFI16 to modulate ChREBP expression during HCMV infection. For this purpose, we measured the mRNA levels of ChREBPα, the canonical ChREBP isoform (29), in WT and IFI16 KO cells at 6 and 24 hpi. At the 6-h time point, ChREBPα mRNA expression levels remained basically unchanged in both cell lines. In contrast, after 24 h of HCMV infection, we recorded a 6-fold induction of ChREBPα expression in cells lacking IFI16, which was almost three times as high as that seen in WT cells that were similarly infected (Fig. 2A). Furthermore, immunofluorescence of HCMV-infected cells at 24 hpi showed enhanced ChREBP nuclear localization (Fig. 2B), indicating that, in response to HCMV infection, ChREBP translocates from the cytoplasm to the nucleus, where it normally acts as a transcription factor (29–31). In particular, the nuclear signal of ChREBP was significantly enhanced in HCMV-infected IFI16 KO cells (Fig. 2C). Thus, IFI16 appears to downregulate ChREBP activity during HCMV infection.

### IFI16 selectively interacts with ChREBP.

**FIG 3:**
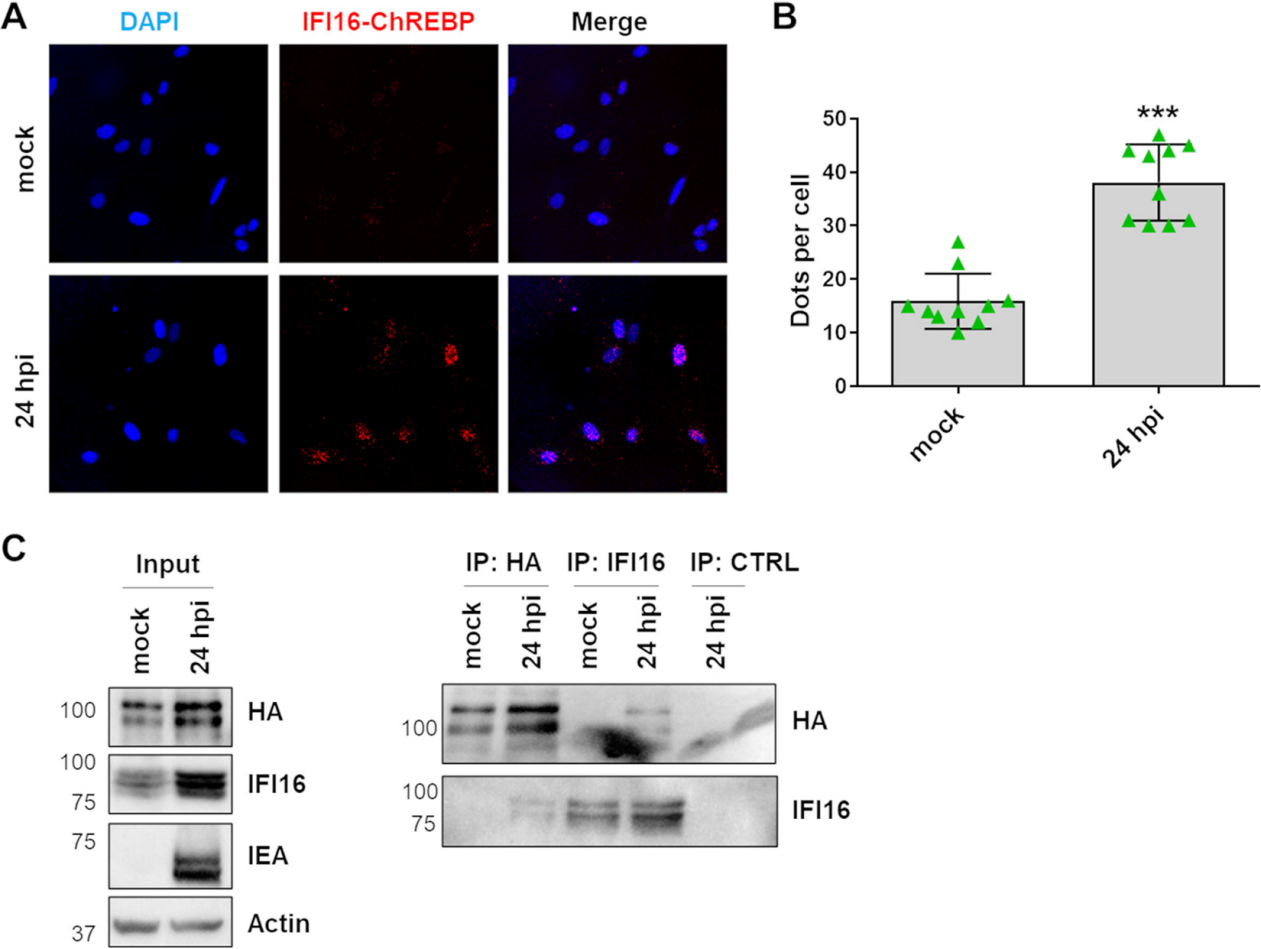
Interplay between ChREBP and IFI16. (A) Representative confocal images of *in situ* PLA between IFI16 and ChREBP in HFFs infected with HCMV (MOI, 1) for the indicated time. The signal was detected as distinct fluorescent dots in the Texas Red channel. Cell nuclei were visualized by DAPI (blue). (B) PLA dots per cell were counted in 5 cells from each sample using ImageJ software and plotted as means and SEM from two independent experiments (***, *P < *0.001; unpaired *t* test). (C) Immunoprecipitation (IP) from HFF lysates. HFFs were transiently transfected with a ChREBP-HA-tagged plasmid and, 24 h later, infected with HCMV (MOI of 1). IPs were performed at 24 hpi using antibodies against an HA tag, IFI16, or the appropriate control antibody (CTRL). Immunoprecipitated and nonimmunoprecipitated (input) proteins were detected by immunoblot analysis using antibodies against IFI16 and HA-tag. Antibodies against actin and HCMV IEA were used as loading and infection controls, respectively.

To better understand how IFI16 affected ChREBP activity, we next examined the possibility that IFI16 could form a complex with ChREBP that would interfere with ChREBP-mediated GLUT4 transcriptional activation. To this end, we measured IFI16/ChREBP interaction in HCMV-infected HFFs by *in situ* proximity ligation assay (PLA). This assay provides a positive signal or dot on confocal pictures when the distance between two molecules is less than 40 nm (32). When we performed PLA on cell cultures at 24 hpi using both anti-ChREBP and anti-IFI16 antibodies, we noticed an increased number of dots per cell in HCMV-infected versus mock-infected HFFs (Fig. 3A and B), indicating close proximity between the two proteins. To further assess the specificity of the IFI16/ChREBP interaction, we performed coimmunoprecipitation (co-IP) experiments using HFFs that had been transiently transfected with a vector carrying a hemagglutinin (HA)-tagged human ChREBP protein and then infected with HCMV for 24 h. As shown in Fig. 3C, IFI16 was detectable in lysates from HCMV-infected cells immunoprecipitated with the anti-HA antibody but not in the negative control. Moreover, reciprocal IPs corroborate these results, establishing that IFI16 selectively interacts with ChREBP during HCMV infection.

### GLUT4 promoter activation is enhanced in the absence of IFI16.

**FIG 4:**
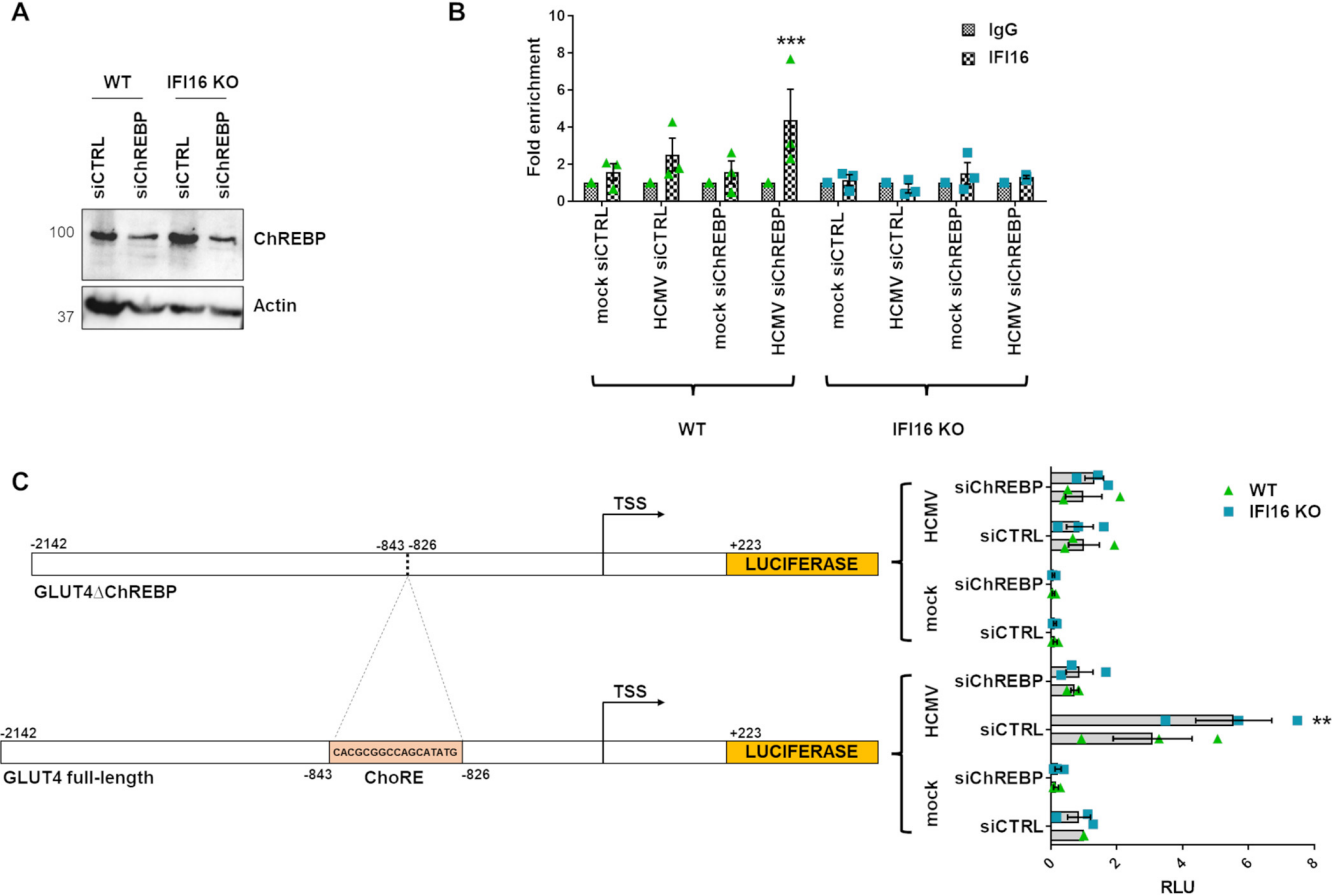
Modulation of GLUT4 promoter activation by IFI16. (A) Efficiency of ChREBP depletion assayed by Western blotting using a polyclonal antibody against human ChREBP. Actin was used as a loading control. (B) WT and IFI16 KO HFFs were electroporated with a mixture of 3 different small interfering RNAs targeting ChREBP (siChREBP) or scrambled control siRNA (siCTRL) and then infected with HCMV at an MOI of 1. After 24 h, cells were cross-linked with formaldehyde, and then IFI16 immune complexes were isolated by immunoprecipitation. Purified ChIP DNA was analyzed by qPCR for the presence of the GLUT4 DNA promoter sequence. The control antibody (IgG) baseline was arbitrarily set at a value of 1. Bars show means and SEM from three independent experiments (***, *P < *0.001; two-way ANOVA followed by Bonferroni’s posttests, for comparison of IFI16 antibody versus IgG control). (C) Schematic model of GLUT4 promoter (nucleotides −2142 to +223). The ChoRE-responsive site is shown (nucleotides −843 to −826). The transcription start site (TSS) is marked by the black arrow. WT and IFI16 KO HFFs were electroporated with siChREBP or siCTRL and with luciferase reporter plasmids containing either the full-length GLUT4 promoter or a promoter harboring a ChoRE site deletion (GLUT4ΔChREBP). Cells were infected with HCMV (MOI, 1) or left uninfected, and luciferase activity was assayed after 24 h. Luciferase activity in whole-cell lysates was normalized to *Renilla* luciferase activity. The mean value of siCTRL-WT mock-infected cells was arbitrarily set to 1. Bars show means and SEM from three independent experiments (**, *P < *0.01; two-way ANOVA followed by Bonferroni’s posttests, for comparison of WT versus IFI16 KO cells).

To ascertain whether IFI16-driven GLUT4 transcriptional regulation requires ChREBP, we first sought to assess the association of IFI16 with the carbohydrate response element (ChoRE) motif of the GLUT4 promoter in HCMV-infected HFFs in the presence or absence of ChREBP. To this end, WT or IFI16 KO HFFs were first silenced for ChREBP expression through specific small interfering RNAs (siRNAs) (siChREBP) or scrambled control (siCTRL) and then infected with HCMV (MOI, 1) for 24 h. As shown in Fig. 4A, ChREBP expression levels were efficiently inhibited in siChREBP- versus siCTRL-treated cells. Cell extracts were then cross-linked with formaldehyde, sonicated, and subjected to chromatin immunoprecipitation (ChIP) using anti-IFI16 or anti-IgG polyclonal antibodies (28). The DNA released from the immunocomplexes was then analyzed by quantitative PCR (qPCR) for the presence of GLUT4. As shown in Fig. 4B, the GLUT4 DNA promoter was detectable in the IFI16-specific immune complexes from HCMV-infected WT but not IFI16 KO HFFs. In particular, a significant enhancement was seen in HCMV-infected siChREBP WT cells. As expected, we did not detect GLUT4 DNA promoter in uninfected WT or IFI16 KO HFFs.

To further define the functional role of IFI16 on GLUT4 promoter activity, we used two luciferase reporter plasmids driven by the full-length GLUT4 gene promoter or by a mutant lacking the ChREBP responsive element (from −843 to −826, named ChoRE) (GLUT4ΔChREBP) (Fig. 4C).

To understand if HCMV-mediated modulation of GLUT4 expression requires ChREBP and/or IFI16, cells were again silenced for ChREBP (siChREBP) or scrambled control (siCTRL) and electroporated with luciferase reporter plasmids. WT and IFI16 KO HFFs were then infected with HCMV or left uninfected (mock infected). Luciferase activity was then assessed after 24 h of incubation.

As shown in Fig. 4C, we observed a robust induction of luciferase activity in cells transfected with the GLUT4 construct and infected with HCMV for 24 h, while cells transfected with the GLUT4ΔChREBP reporter plasmid displayed a reduced increase in luciferase activity, indicating that the ChoRE site is crucial for GLUT4 transcriptional activation by HCMV. HCMV-infected IFI16 KO cells transfected with the GLUT4 construct for 24 h showed a much higher luciferase activity than WT cells (∼2-folds), underscoring a robust inhibitory activity of IFI16 on the GLUT4 promoter. Importantly, siChREBP-treated HFFs were not responsive to HCMV-mediated GLUT4 promoter activation, regardless of the presence of IFI16, suggesting that ChREBP is required for GLUT4 promoter activation.

### IFI16 limits HCMV-mediated cholesteryl ester accumulation.

ChREBP is a transcription factor known to activate the transcription of lipogenic enzymes upon HCMV infection in a sterol regulatory element-binding protein (SREBP)-independent way (29). The transcriptional targets of ChREBP include the *de novo* lipogenesis (DNL) genes acetyl-coenzyme A (CoA), carboxylase 1 (ACC1), and fatty acid synthase (FAS), each containing a ChoRE element in their promoter/regulatory region (33, 34).

**FIG 5:**
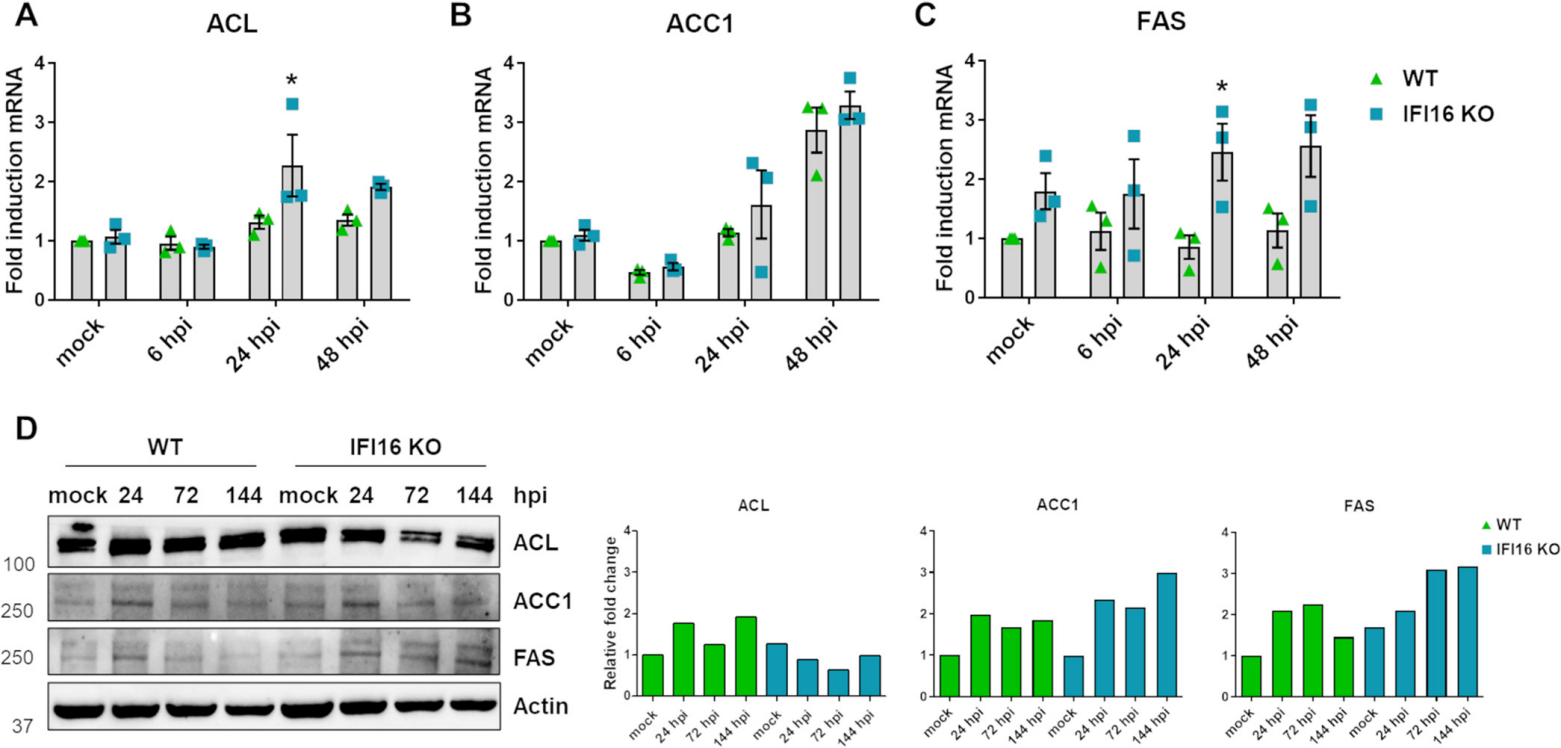
Effects of IFI16 silencing on HCMV-induced lipogenesis. IFI16 KO and WT HFFs were infected with HCMV (MOI 1). At 6, 24, and 48 hpi, total RNA was isolated and subjected to RT-qPCR to measure mRNA expression levels of the lipogenic enzymes ACL (A), ACC1 (B), and FAS (C). Values were normalized to the housekeeping gene GAPDH mRNA and plotted as fold induction relative to mock-infected WT cells (set at 1). Bars show means and SEM from three independent experiments (*, *P < *0.05; two-way ANOVA followed by Bonferroni’s posttests, for comparison of WT versus IFI16 KO cells). (D) Western blot analysis of protein lysates from uninfected (mock) or infected cells using antibodies against ACL, ACC1, FAS, or actin. One representative blot (left) and densitometric analysis (right) are shown. Values are expressed as fold change in ACL, ACC1, and FAS expression normalized to actin.

As ChREBP translocation to the nucleus induces lipogenic enzyme transcription, and HCMV infection increases *de novo* fatty acid synthesis through transactivation of lipogenic enzymes (35, 36), we asked whether IFI16 would also play an inhibitory role in these processes. To answer this question, we first analyzed mRNA and protein expression of ATP-citrate lyase (ACL), ACC1, and FAS in HCMV-infected IFI16 KO and WT cells. As shown in Fig. 5, mRNA and protein expression levels of FAS were upregulated in either mock or HCMV-infected IFI16 KO compared to similarly treated WT HFFs (Fig. 5C and D). A similar trend, albeit less pronounced, was also observed for ACC1 (Fig. 5B to D), while ACL induction was not affected by the presence of IFI16 (Fig. 5A to D). Thus, these results support the hypothesis that IFI16 may play an inhibitory role in HCMV-induced lipogenesis.

To verify whether the differences in lipogenic enzyme expression between WT and IFI16 KO HFFs corresponded to differences in lipid composition, we performed lipidomic analysis by reversed-phase liquid chromatography coupled with quadrupole-time-of-flight mass spectrometry (RP-LC-Q-TOF-MS). For these experiments, cells were grown to confluence in serum-free medium to limit lipid or metabolite contamination from the bovine serum (19, 24).

**FIG 6:**
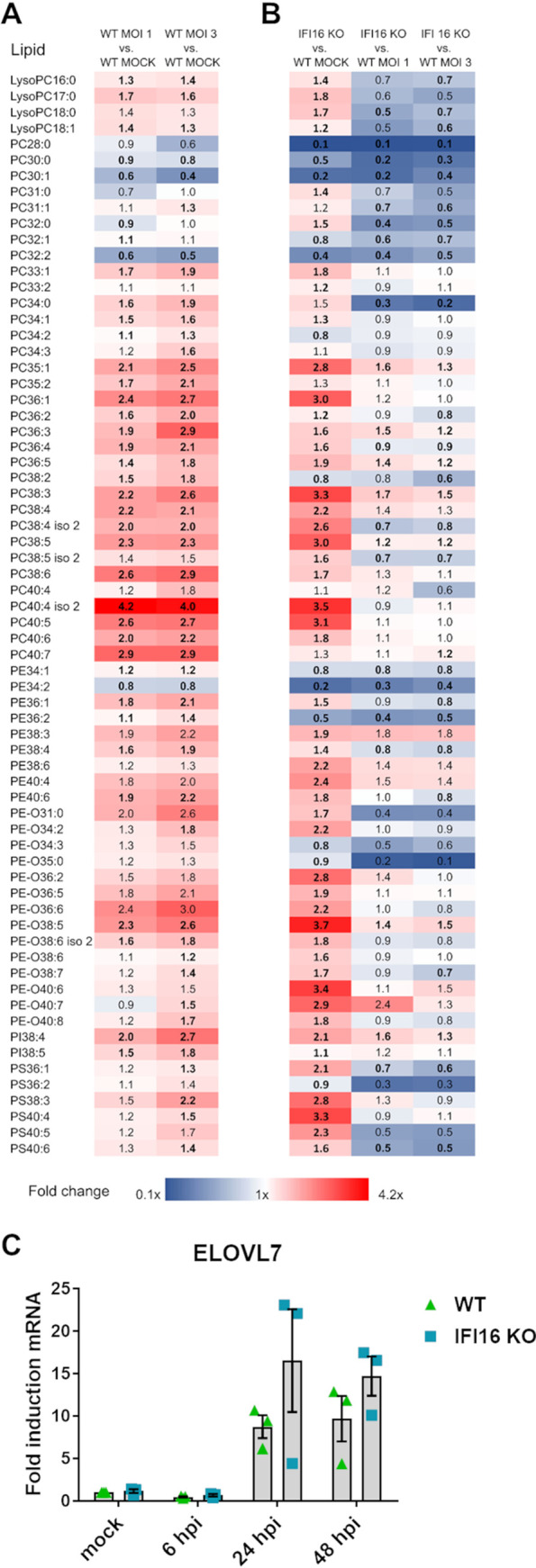
Lipidomic comparison of WT and IFI16 KO HFFs. WT and IFI16 KO HFF cells were infected with HCMV (MOI of 1 or 3) or left uninfected (mock) and maintained in serum-free medium. At 96 hpi, total lipids from cells were extracted and analyzed by LC-MS/MS. The fold changes of lipid species peak area are visualized as a heat map showing the levels of upregulated (red, fold change > 1) and downregulated (blue, fold change < 1) glycerophospholipids. Boldface shows statistically significant values (Mann-Whitney unpaired test, *P < *0.05). PC, glycerophosphocholine; PE, glycerophosphoethanolamine; PE-O, ether analogue of glycerophosphoethanolamine; PS, glycerophosphoserine, PI, glycerophosphoinositol. *n *=* *2 independent determinations. (A) Infected WT (MOI of 1 [left] and 3 [right]) versus uninfected HFFs. (B) IFI16 KO versus WT HFFs, mock (left), and HCMV-infected IFI16 KO versus HCMV-infected WT HFFs (MOI of 1 [middle] and 3 [right]). (C) WT and IFI16 KO HFFs were infected with HCMV (MOI, 1), and at 6, 24, and 48 hpi, total RNA was isolated and subjected to RT-qPCR to measure mRNA expression levels of the fatty acid elongase 7 (ELOVL7). Values were normalized to the housekeeping gene GAPDH mRNA and plotted as fold induction relative to WT mock-infected cells (set at 1). Bars show means and SEM from three independent experiments (not significant; two-way ANOVA followed by Bonferroni’s posttests, for comparison of WT versus IFI16 KO cells).

First, we estimated the amounts of the phospholipids with very-long-chain-fatty-acid tails (PL-VLCFAs) in HCMV-infected versus uninfected WT HFFs. As shown in Fig. 6A, PL-VLCFAs were generally increased in HCMV-infected WT cells compared to uninfected cells, in good agreement with previous results by Purdy et al. (24).

When we compared the MS patterns of the various glycerophospholipid species, we noticed that the lipid fingerprint of IFI16 KO HFFs was different from that of WT cells, even though not all lipid classes were equally affected (Fig. 6B, left column). The majority of tested glycerophospholipids were upregulated in uninfected IFI16 KO cells in comparison with uninfected WT cells. However, we observed that some lipid species were downregulated (e.g., glycerophosphocholines [PCs] and glycerophosphoethanolamines [PEs]), in particular lipids species containing medium-chain fatty acids with up to 2 double bonds, such as PC_28:0_, PC_30:0_, and PC_30:1_.

The lipid types that were most significantly modulated upon infection between IFI16 KO and WT cells are listed in Fig. 6B (middle and right columns). Interestingly, a major fraction of long-chain and unsaturated (37) PCs, PEs, and glycerophosphoinositols (PIs) (e.g., PE_38:6_, PE_38:3_, and PI_38:4_) were upregulated in infected IFI16 KO cells with respect to WT cells. Finally, lipids containing medium-chain fatty acid substituents with up to 2 double bonds (e.g., PC_32:0_, PC_32:1_, and PC_34:0_), lysoglycerophosphocholines (lysoPCs), and some diacylglycerolphosphoserines (PSs) were significantly downregulated in IFI16 KO versus WT HFFs following HCMV infection.

Given that HCMV upregulates host fatty acid elongase 7 (ELOVL7) expression to catalyze fatty acid elongation (24), we decided to investigate whether IFI16 would also play a role in ELOVL7 gene expression. Interestingly, we observed a slight upregulation in ELOVL7 expression in cells depleted of IFI16 and infected with HCMV, indicating that IFI16 protein is partially responsible for HCMV-induced ELOVL7 expression (Fig. 6C) (38).

**FIG 7:**
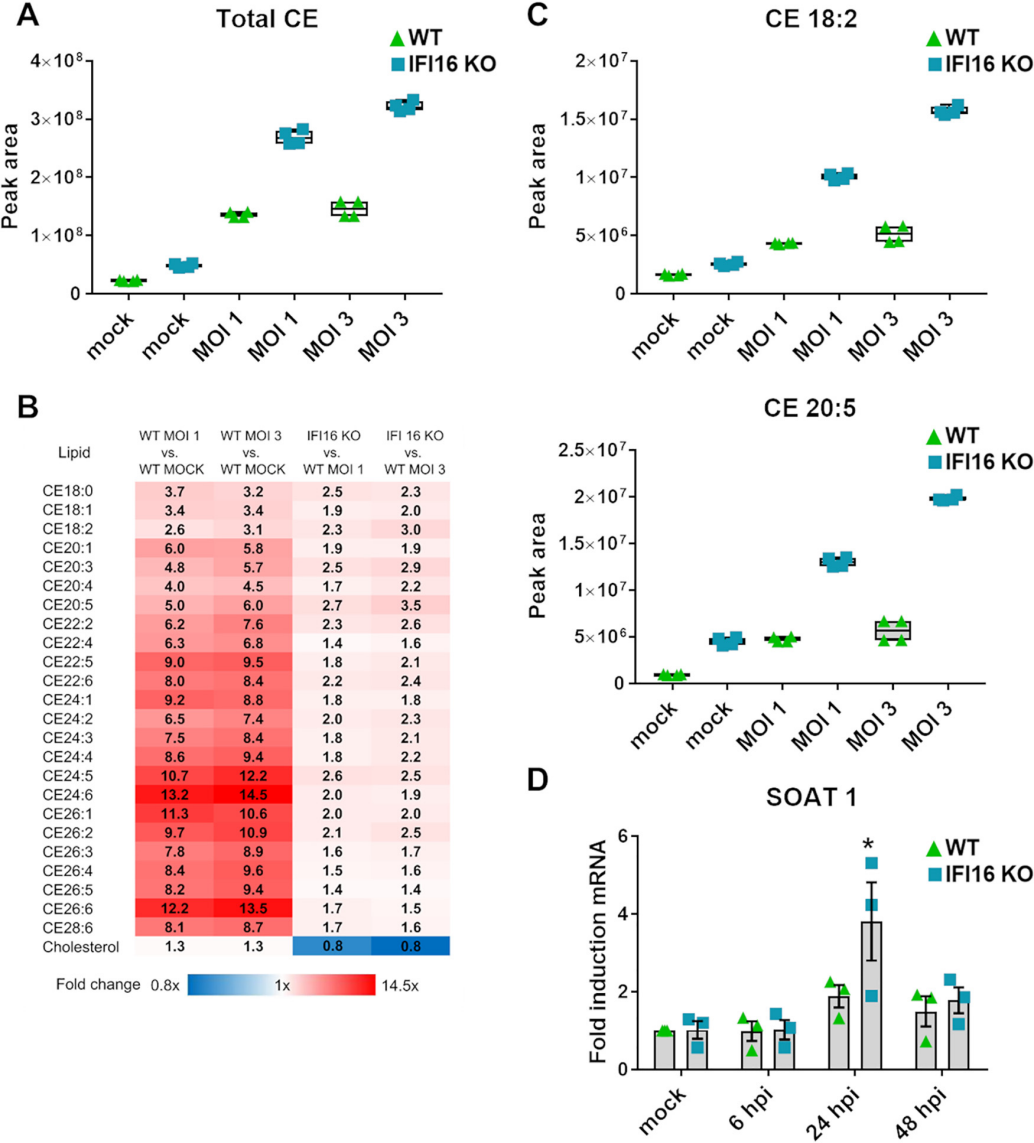
IFI16 is partially responsible for HCMV-mediated CE accumulation. CE comparison in mock- or HCMV-infected IFI16 KO versus WT HFFs. Cells were infected with HCMV (MOI of 1 or 3) and maintained in serum-free medium. At 96 hpi, total lipids from cells were extracted and analyzed by LC-MS/MS. (A) Comparison of total CE content measured as the sum of the peak area for all CE species. (B) Heat map of CE species showing the levels of upregulated (red, fold change > 1) and downregulated (blue, fold change < 1) CE species in HCMV-infected relative to uninfected HFFs (two left panels) and HCMV-infected IFI16 KO relative to HCMV-infected WT cells (two right panels). Boldface shows statistically significant values (nonparametric Student's *t* test, *P < *0.05). (C) Levels—measured by the peak area—of the significantly modulated CE species in HCMV-infected or uninfected WT and IFI16 KO HFFs. Center lines show the medians; box limits indicate the 25th and 75th percentiles as determined by R software; whiskers extend 1.5 times the interquartile range from the 25th and 75th percentiles; crosses represent sample means. *n *=* *2 independent determinations. (D) WT and IFI16 KO HFFs were infected with HCMV (MOI, 1). At 6, 24, and 48 hpi, total RNA was isolated and subjected to RT-qPCR to measure SOAT1 expression levels. Values were normalized to GAPDH mRNA and plotted as fold induction relative to WT mock-infected cells (set at 1). Bars show means and SEM from three independent experiments (*, *P < *0.05; two-way ANOVA followed by Bonferroni’s posttests, for comparison of WT versus IFI16 KO cells).

Due to the relevance and implications of cholesterol esters (CEs) for viral replication (39–42), we also measured CE accumulation in IFI16 KO versus WT HFFs. The fold change (FC) difference in total CE lipids between HCMV-infected IFI16 KO and WT HFFs is shown in Fig. 7A (FC = 2.2 [*P* < 0.001] at an MOI of 3 and 2.0 [*P* < 0.001] at an MOI of 1). Interestingly, all CEs were significantly upregulated (*P < *0.005) in IFI16 KO cells in comparison with WT at both MOI (Fig. 7B). The most affected CE species were CE_20:5_ (FC = 2.7 for an MOI of 1 and 3.5 for an MOI of 3) and CE_18:2_ (FC = 2.3 for an MOI of 1 and 3.0 for an MOI of 3) (Fig. 7C).

Intracellular CE biosynthesis is mediated by sterol *O*-acyl transferase 1 (SOAT; also known as acyl-CoA:cholesterol acyltransferase [ACAT]). To determine whether SOAT1 gene expression was affected by HCMV replication and/or IFI16 sensor activity, we examined its mRNA expression pattern during a time course of HCMV infection of WT and IFI16 KO HFFs. As shown in Fig. 7D, SOAT1 gene expression increased upon HCMV infection, confirming previous studies (43). Importantly, SOAT1 reached a 2-fold-greater expression in IFI16 KO than WT infected cells, reflecting the lipidomic analysis (Fig. 7D).

Taken together, these results support the notion that HCMV can reprogram the cellular lipid environment through modulation of lipid metabolic enzymes and reveal a novel functional role of IFI16 in this process.

### IFI16 affects HCMV infectivity.

**FIG 8:**
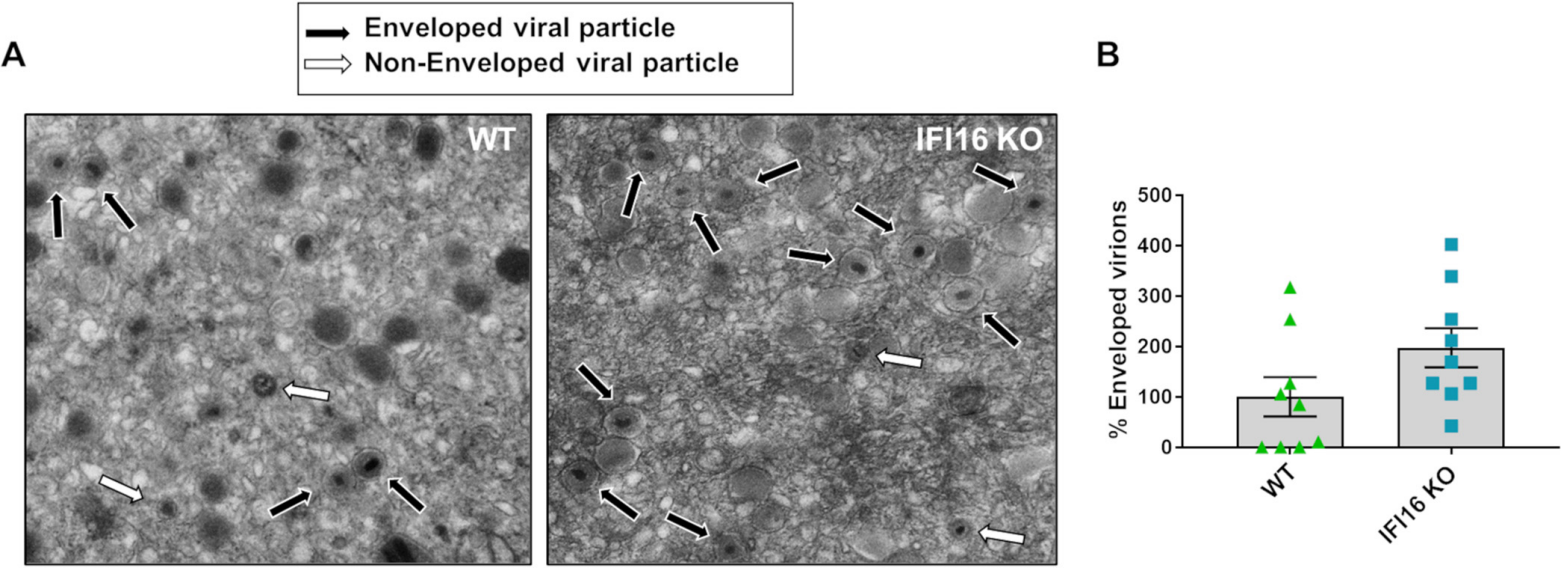
Transmission electron micrographs of HCMV-infected WT and IFI16 KO HFFs. (A) Monolayers were infected with HCMV (MOI, 1) and processed for electron microscopy at 7 dpi. Multiple frames from each sample were imaged and photographed. (Left) WT; (right) IFI16 KO. Particles from a representative cell are shown. White and black arrows indicate representative nonenveloped and enveloped particles, respectively. (B) The number of enveloped particles was counted on 9 frames from different cells and plotted as fold induction relative to WT-infected cells (set at 100%). Data are mean percentages of the enveloped particles and SEM (not significant; unpaired *t* test).

In addition to being a source of energy, enhanced fatty acid biosynthesis is crucial for HCMV budding because it increases the amount of the main constituents necessary for viral envelope formation (37, 44). To explore the role of IFI16 in this process, we first quantified the amount of viral enveloped particles—i.e., double-layered particles formed by tegumentation and secondary envelopment in the secretory pathway—in the cytoplasm of WT versus IFI18 KO HFFs (Fig. 8A). Transmission electron microscopy (TEM) revealed that the number of enveloped particles was reduced in the cytoplasm of WT HFFs in comparison with IFI16 KO HFFs (Fig. 8B), suggesting that IFI16 diminishes virion maturation by subverting HCMV-induced reprogramming of lipid metabolism. Of note, these results are in line with previous findings from our lab (25) showing that IFI16 restricts HCMV replication (25, 28).

**FIG 9:**
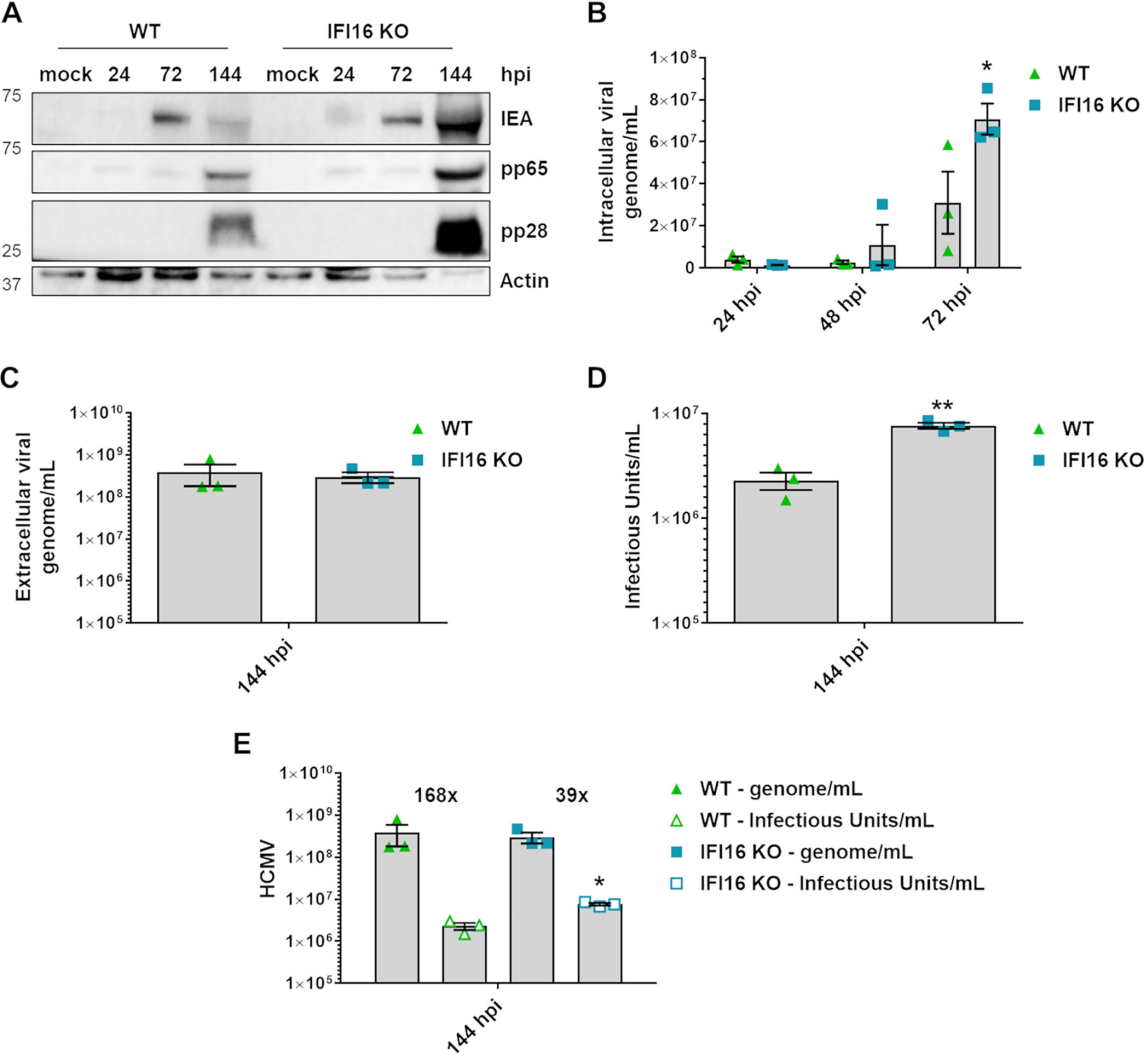
IFI16 impairs the infectivity of HCMV viral particles. (A) WT and IFI16 KO HFFs were infected with HCMV (MOI, 0.5). At the indicated time points, cells were harvested and subjected to Western blot analysis using monoclonal antibodies against HCMV IEA, pp65, pp28, or actin as the loading control. (B) WT and IFI16 KO HFFs were infected with HCMV (MOI, 0.5). At the indicated time points, the DNA was extracted from infected cells, and the number of HCMV genomes was measured by qPCR. The primers amplified a segment of the IE1 gene to determine the number of viral DNA genomes per nanogram of cellular reference DNA (GAPDH gene). Bars represent the means and SEM from three independent experiments (*, *P < *0.05; two-way ANOVA followed by Bonferroni’s posttests, for comparison of WT versus IFI16 KO cells). (C) The number of HCMV genomes released in the supernatants of infected cells was measured by qPCR (not significant; unpaired *t* test). (D) The supernatants used for panel C were used to determine the number of infectious units/mL by virus yield assay. Bars represent means and SEM from three independent experiments (**, *P < *0.01; unpaired t test). (E) The genome-to-infectious unit ratio was determined, and the values are above the bars (*, *P < *0.05, unpaired *t* test).

Finally, to support and complement the EM results and to further strengthen the role of IFI16 in modulating lipid reprogramming, we assessed the role of IFI16 in restricting HCMV replication in our stable KO model under serum-free conditions. For this purpose, we assessed the accumulation of viral proteins in HCMV-infected WT versus IFI16 KO HFFs at a low MOI (0.5). As shown in Fig. 9A, we observed reduced expression of viral proteins in cells harboring IFI16, confirming our previous findings that IFI16 restricts HCMV replication (25). Consistently, the intracellular number of HCMV genome copies at 48 and 72 hpi was increased in IFI16 KO cells compared to WT cells (Fig. 9B), supporting a role of IFI16 in suppressing viral replication (25).

This increase in intracellular viral genomes in IFI16 KO cells was not reflected by the total number of HCMV genomes released into the supernatants at later times (Fig. 9C), which was similar for IFI16 KO and WT cells. To explain this discrepancy, we measured the release of infectious progeny. Surprisingly, as reported in Fig. 9D, IFI16 KO cells released infectious HCMV progeny with an increased infectivity than similarly infected WT HFFs. The ratio of HCMV genomes to infectious units (i.e., genome-to-IU ratio) was approximately 168× for WT HFF, whereas it was 39× for IFI16 KO cells (Fig. 9E). Because a higher genome-to-IU ratio implies lower particle infectivity, IFI16 KO cells released four times more infectious viral particles, suggesting that IFI16 impairs HCMV-induced lipogenesis and thus impairs maturation and release of infectious viral particles.

Altogether, our findings support a model where IFI16 not only restricts HCMV replication but also can curb virion infectivity by acting as a metabolism regulator.

## DISCUSSION

The interplay between HCMV and the host cell metabolism is a crucial aspect of this virus’ life cycle, as its replication largely depends on the energy and biosynthetic precursors supplied by the infected cell (10, 38). In this regard, glucose uptake has been shown to be significantly increased in HCMV-infected cells (16, 45), corroborating the evidence that HCMV, like other viruses, exploits glucose metabolism for its own benefit.

Glucose uptake mainly involves specific carriers belonging to the family of glucose transporters (GLUTs). There are 13 different GLUTs in mammalian cells, GLUT1 to -12 plus the proton (H^+^)-myoinositol cotransporter (HMIT) (46, 47). Structurally, GLUTs can be divided into three classes: GLUT1 to -4 (class I); GLUT5, -7, -9, and -11 (class II); and GLUT6, -8, -10, and -12 and HMIT (class III) (46, 47), each with different tissue specificities and affinities for glucose. For example, in HCMV-infected human fibroblasts, the HCMV IE72 protein can simultaneously suppress GLUT1 mRNA and induce GLUT4, which has a greater glucose transport capacity (20). This switching of glucose transporters appears to be essential for successful HCMV infection and replication, as inhibition of glucose uptake via GLUT4 by indinavir impedes viral production (20).

In the course of evolution, HCMV has evolved a number of strategies aimed at the modulation of host metabolic pathways to favor virion production. Conversely, the host has long tried to counteract this hijacking threat through innate immunity. In this regard, we have previously demonstrated that IFI16 acts as a cellular restriction factor of HCMV replication (25) and plays a key role in HCMV-related innate immunity (26, 28).

**FIG 10:**
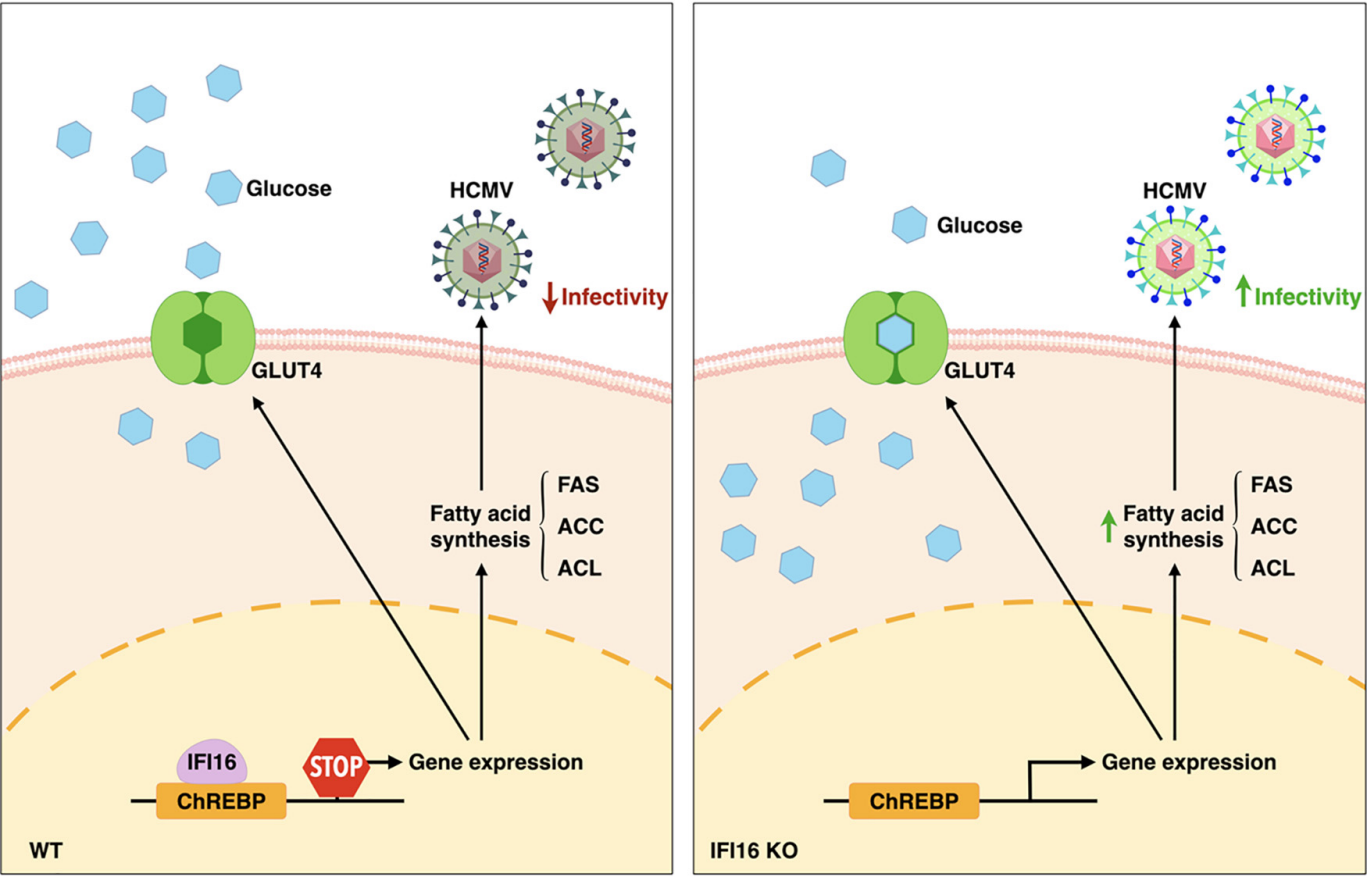
Proposed model of IFI16 control on HCMV-induced metabolic changes. Upon HCMV infection, IFI16 forms an inhibitory complex with the transcription factor ChREBP, thereby downmodulating the expression of GLUT4 and reducing glucose consumption. Furthermore, IFI16-ChREBP cooperation leads to decreased expression of genes involved in HCMV-induced lipogenesis, which leads to impaired lipid synthesis and reduced infectivity of viral particles.

In the present study, we add another layer of complexity to the restriction activity of IFI16 by showing that this pathogen sensor can counteract HCMV-induced metabolic reprogramming, as summarized schematically in Fig. 10. In particular, we show that IFI16 decreases HCMV-induced glucose uptake through inhibition of GLUT4 mRNA expression in HFFs, leading to decreased glucose uptake and consumption. We also show that GLUT4 inhibition relies on the cooperation between IFI16 and ChREBP, which form a complex that inhibits HCMV-induced transcriptional activation of the GLUT4 promoter in HFFs.

ChREBP is a key regulator of lipogenic gene expression. In synergy with SREBP, it can in fact regulate the expression of genes involved in the conversion of glucose to fatty acids and nucleotides. Furthermore, ChREBP deficiency has been shown to impair glucose uptake and glycolysis in HCMV-infected cells, thereby decreasing the glucose-carbon flux for anabolic processes, such as lipid synthesis, NADPH generation, and nucleotide biosynthesis (21, 48). Here, we unveil an unprecedented selective interaction between IFI16 and ChREBP to modulate GLUT4 expression during HCMV infection, resulting in decreased glycolytic and lipogenic gene expression.

We also report an overall increase in lipid concentration, especially of CE species, in HCMV-infected IFI16 KO versus WT HFFs, indicating that IFI16 is a negative regulator of lipogenesis. However, the fact that metabolic reprogramming still occurs in HCMV-infected WT HFFs despite the presence of IFI16 indicates that while IFI16 may play an important role in lipid transcriptional downregulation, its activity is not sufficient to completely overcome the metabolic changes induced by HCMV. Nevertheless, our findings are in line with the notion that herpesviruses rely on host lipid metabolism to replicate, which is also supported by our lipidomic analysis of HCMV-infected cells.

The finding of CE accumulation upon HCMV infection did not come as a surprise, as it had already been reported by Fabricant et al. in the early 1980s while they were studying other herpesviruses (40). In contrast, in a more recent study by Low et al. (49), no CE lipid accumulation in HCMV-infected fibroblasts was observed. This discrepancy may be ascribable to the presence of fetal bovine serum (FBS)—and therefore lipids—in the growth medium, which may have led to bias in the normalization step. Interestingly, previous screenings revealed that increased CE levels may also occur in cells infected with RNA viruses, such as HCV (42, 50). Accordingly, SOAT1, responsible for intracellular CE biosynthesis from cholesterol and fatty acyl-CoA, was shown to be upregulated early upon HCMV infection, and its inhibition restrained viral replication (43). Our findings of increased CE levels in HCMV-infected IFI16 KO cells, which are further accompanied by more robust SOAT1 upregulation than in similarly infected WT HFFs, support our hypothesis that IFI16 is a negative regulator of lipid synthesis.

The final outcome of IFI16 restriction activity on HCMV enhanced metabolic pathways is a significant and highly reproducible decrease in the release of infectious virions. HCMV virion assembly is a complex process involving initial envelopment at the inner nuclear membrane and de-envelopment at the outer nuclear membrane to release partially tegumented nucleocapsids into the cytoplasm (44, 51). There, nucleocapsids obtain their envelope in a secondary envelopment process by budding into vesicles of the secretory pathway prior to their release into supernatant (52). In this context, our electron microscopy experiments show no obvious defect in virion morphogenesis between WT and IFI16 KO cells, but we found that the number of enveloped nucleocapsids is slightly decreased in WT HFFs. It is reasonable to assume that the reduction in viral protein expression and restriction of viral replication in WT HFFs compared with IFI16 KO cells results in the production of fewer viral particles, which explains our electron-microscopic observations. The IFI16-dependent restriction of viral replication shown in this work is in complete agreement with our previous results (25). Despite the difference intracellular virion number, the release of viral particles from infected cells did not appear to be affected, as there was no difference in extracellular genome copy number between WT and IFI16-KO cells. The infectivity of released virus particles, however, was decreased by a factor of 4 in WT-HFF. This may well be related to IFI16 restriction activity on the lipid metabolic pathway, since selective impairment of lipid metabolism, e.g., by knockdown of ELOVL7 or PERK, was associated with reduced infectivity of virus particles in previous work (14, 24).

Together, these results not only confirm IFI16 as a restriction factor of HCMV replication, but also identify IFI16 as a metabolism regulator affecting virion infectivity.

Another implication of our findings involves the potential interplay among lipid metabolic disorders, HCMV, and atherosclerosis, whose pathogenetic mechanism is not fully understood (53). In this regard, the possible infectious etiology of atherosclerosis has recently gained increasing attention (54–56), with HCMV being proposed as the main culprit (53, 57, 58). Indeed, several studies have shown a correlation between HCMV seropositivity and increased risk of coronary atherosclerosis (59, 60), and HCMV DNA and viral protein have been detected in atherosclerotic lesions (61). Moreover, animal models have provided evidence that HCMV infection promotes atherogenesis (62). Last, CE metabolism has long been linked to the development of atherosclerosis (63, 64). Thus, based on the results of this study, experiments are ongoing to explore the potential contribution of HCMV-driven metabolic reprogramming to the pathogenesis of atherosclerosis.

Altogether, our findings strengthen the idea that IFI16 is a global regulator of HCMV-induced modulation of lipid metabolism, which may pave the way to the discovery of new therapeutic targets for HCMV therapy.

## MATERIALS AND METHODS

### Cells and viruses.

Primary HFFs (American Type Culture Collection; ATCC SCRC-1041) were cultured in Dulbecco’s modified Eagle’s medium (Sigma-Aldrich) supplemented with 10% fetal calf serum (FCS; Sigma-Aldrich) according to ATCC specifications. HFFs with IFI16 silenced (IFI16 KO) were generated by the CRISPR/Cas9 system, as previously described (27, 65). The HCMV laboratory strain AD169 (ATCC-VR538) was propagated and titrated by standard plaque assay (25). Prior to infection, cells were maintained at full confluence for 24 h in serum-containing growth medium and switched to serum-free medium the day before the infection. Infections were performed at a MOI of 1 or 3 infectious units per cell, depending on the experiment.

### Plasmids.

The GLUT4 promoter was kindly provided by K. T. Dalen (University of Oslo, Norway) and generated according to Dalen et al. (66). The plasmid expressing HA-tagged human ChREBPα was kindly provided by Y. Yu (University of Pennsylvania, Philadelphia, PA, USA) (21). The mutant construct of the GLUT4 promoter lacking the ChREBP binding site (GLUT4ΔChREBP) was cloned starting from a pGL3-Basic vector carrying the GLUT4 promoter previously described, using the following primers: GLUT4ΔChREBP Fw, AGGATCCAAACCCGGAGCAGCCTCCAGAGAGCGTGTCGTTCTCAGAGACCTCAGAGGCTC; GLUT4ΔChREBP Rv, CCACCAGCCCTGAGGTCTCTGAGCCTCTGAGGTCTCTGAGAACGACACGCTCTCTGGAGG. Mutations were confirmed by DNA sequencing. The IE1-encoding plasmid pSGIE72 was used in the quantitative nucleic acid analysis.

### RNA isolation and semiquantitative RT-qPCR.

Total RNA was extracted using TRI Reagent solution (Life Technologies) according to the manufacturer’s instructions, and 1 μg was retrotranscribed using the Revert-Aid H-Minus FirstStrand cDNA synthesis kit (Thermo Fisher Scientific). Comparison of mRNA expression between samples (i.e., WT versus IFI16 KO) was performed by SYBR green-based reverse transcription-qPCR (RT-qPCR) using an Mx3000P apparatus (Stratagene) with the following primers: GLUT4 Fw, GGAGCTGGTGTGGTCAACACA; GLUT4 Rv, GGAGCAGAGCCACAGTCATCA; ACL Fw, TGTAACAGAGCCAGGAACCC; ACL Rv, CTGTACCCCAGTGGCTGTTT; ACC1 Fw, TTAACAGCTGTGGAGTCTGGCTGT; ACC1 Rv, AACACTCGATGGAGTTTCTCGCCT; FAS Fw, AGGCTGAGACGGAGGCCATA; FAS Rv, AAAGCTCAGCTCCTGGCGGT; CHREBPα Fw, AGTGCTTGAGCCTGGCCTAC; CHREBPα Rv, TTGTTCAGGCGGATCTTGTC; ELOVL7 Fw, GGCCAGCCTACCAGAAGTATTTG; ELOVL7 Rv, GGCGACAATAACAAACTGGACAAG; SOAT1 Fw, CCACTGGTCCAGATGAGTTTAG; SOAT1 Rv, GGGAACATGCAGAGTACCTTT; GAPDH (the housekeeping gene encoding glyceraldehyde-3-phosphate dehydrogenase) Fw, AGTGGGTGTCGCTGTTGAAGT; GAPDH Rv, AACGTGTCAGTGGTGGACCTG.

### Inhibition of ChREBP expression.

HFFs were transiently transfected using a MicroPorator (Digital Bio) according to the manufacturer's instructions (1,200 V, 30-ms pulse width, one impulse), with a pool of ChREBP small interfering RNAs (siChREBP; CHREBP [MLXIPL] human siRNA oligonucleotide duplex; OriGene SR309520) or control siRNA (siCTRL) as a negative control, according to the manufacturer’s protocols. siRNA-induced blockade of ChREBP expression was checked by Western blotting.

### DNA extraction and viral load determination.

Intracellular DNA was extracted using TRI Reagent solution (Life Technologies) according to the manufacturer’s instructions. Quantification of intracellular and extracellular HCMV copy numbers was evaluated via quantitative real-time PCR (qPCR) analysis on an Mx 3000 P apparatus (Stratagene), using primers to amplify a segment of the IE1 gene (Fw, 5′-TCAGTGCTCCCCTGATGAGA-3′; Rv, 5′-GATCAATGTGCGTGAGCACC-3′), as described by Biolatti et al. (28). Intracellular HCMV DNA copy numbers were normalized to GAPDH. A standard curve of serially diluted genomic DNA mixed with an IE1-encoding plasmid (from 10^7^ copies to 1 copy) was created in parallel with each analysis.

### Western blot analysis.

Whole-cell protein extracts were prepared and subjected to Western blot analysis as previously described (26, 67). The primary antibodies against the following proteins were used: actin clone C4 (MAB1501; Sigma-Aldrich), α-tubulin (39527; Active Motif), IFI16 (Santo Landolfo, University of Turin, Italy), IFI16 (sc-8023; Santa Cruz Biotechnology), HA (3724; Cell Signaling), immediate early antigen (IEA) (P1215; Virusys), pp65 (CA003; Virusys), and pp28 (P1207; Virusys). Immunocomplexes were detected using appropriate secondary antibodies conjugated with horseradish peroxidase (HRP) (GE Healthcare Europe GmbH) and visualized by enhanced chemiluminescence (Super Signal West Pico; Thermo Fisher Scientific).

### Immunoprecipitation.

Uninfected or HCMV-infected cells (MOI, 1) were washed with 1× phosphate-buffered saline (PBS) and lysed in Triton buffer (50 mM Tris, pH 7.4; 150 mM NaCl; 1 mM EDTA; 1% Triton; protease inhibitors). Proteins (400 μg) were incubated with 4 μg of specific antibodies against IFI16 (Santo Landolfo, University of Turin, Italy) or HA (3724; Cell Signaling), or with rabbit IgG preimmune antibody (NRI01; Cell Sciences) as a negative control, for 2 h at 4°C with rotation, followed by an overnight incubation at 4°C with protein G-Sepharose (Sigma-Aldrich). Immune complexes were collected by centrifugation, washed three times with PBS, resuspended in reducing sample buffer (50 mM Tris, pH 6.8; 10% glycerol; 2% SDS; 1% 2-mercaptoethanol), boiled for 5 min, and resolved on an SDS-PAGE gel to assess protein binding by Western blotting.

### ChIP assay.

ChIP assays were performed using a shearing optimization kit and a OneDay ChIP kit (Diagenode Europe), according to the manufacturer's instructions. Extracts were cross-linked with 1% paraformaldehyde for 15 min and then processed with OneDay ChIP (Diagenode Europe), according to the manufacturer's instructions. DNA fragments were sonicated with a BioruptorH Twin (Diagenode Europe) for 10 cycles (30 s on, 30 s off) on the high-power setting. Immunoprecipitation was performed using 5 μg of anti-IFI16 antibody (Santo Landolfo, University of Turin, Italy). Rabbit IgG was used as a negative control. The DNA solution (1 μL per reaction mixture) was used for qPCR using HCMV- or human-specific primers: ChREBP responsive element Fw, CTCCAGAGAGCGTGTCGTTC; ChREBP responsive element Rv, CGAGGGACAAGTGGTCACAA.

### Luciferase assay.

Cells were electroporated with siChREBP or siCTRL and with a luciferase reporter plasmid driven by the GLUT4 gene promoter and with pRL-SV40 (Promega Italia) plasmid as previously described (25). HFFs were then infected with HCMV (MOI, 1). At 24 hpi, firefly and *Renilla* luciferase activities were measured using the dual-luciferase reporter assay system kit (Promega Italia) and a Lumino luminometer (Stratec Biomedical Systems), as previously described (68). The firefly luciferase activity from the luciferase reporter vector was normalized to that of the *Renilla* luciferase vector (pRL-SV40). Data are expressed as the ratio of relative light units (RLU) measured for firefly luciferase activity to the RLU measured for *Renilla* luciferase activity.

### Immunofluorescence microscopy.

Indirect immunofluorescence analysis was performed as previously described (26). The primary antibodies were those against IEA (P1215; Virusys) and ChREBP (ab92809; Abcam). Signals were detected using goat anti-rabbit or goat anti-mouse conjugated secondary antibodies (Thermo Fisher Scientific). Nuclei were counterstained with 4′,6-diamidino-2-phenylindole (DAPI). Samples were observed using a fluorescence microscope (Olympus IX70) equipped with cellSens standard microscopy imaging software. The percentage of cells showing ChREBP nuclear translocation in IEA-positive nuclei was calculated using ImageJ software.

### Proximity ligation assay.

The PLA was performed using the DuoLink (Sigma-Aldrich) PLA kit to detect protein-protein interactions using fluorescence microscopy according to the manufacturer’s instructions. Briefly, HFFs were cultured and infected with HCMV at an MOI of 1 for 24 h, fixed for 15 min at room temperature with 4% paraformaldehyde, permeabilized with 0.2% Triton X-100, and blocked with 10% HCMV-negative human serum for 30 min at RT. Cells were then incubated with the primary antibodies diluted in Tris-buffered saline (TBS)–0.05% Tween for 1 h, washed, and then incubated for an additional h at 37°C with species-specific PLA probes under hybridization conditions and in the presence of 2 additional oligonucleotides to facilitate the hybridization only in close proximity (40 nm). A ligase was then added to join the two hybridized oligonucleotides, forming a closed circle. Using the ligated circle as the template, rolling-circle amplification was initiated by adding an amplification solution, generating a concatemeric product extending from the oligonucleotide arm of the PLA probe. Last, a detection solution consisting of fluorophore-labeled oligonucleotides was added, and the labeled oligonucleotides were hybridized to the concatemeric products. The signal was detected as distinct fluorescent dots in the Texas Red channel and analyzed by confocal microscope (Leica Microsystem). Negative controls consisted of mock-infected cells that were otherwise treated in the same way as the infected cells.

### Glucose metabolic assays.

For glucose assays, HFFs were plated in 24-well cell plates and cultured in serum-free DMEM. Cells were infected at an MOI of 1 and 3. The culture medium was harvested at 24, 48, 72, and 144 hpi, and the glucose concentration in the culture medium was measured by means of a Biosen C-line analyzer (EKF Diagnostics).

### Electron microscopy.

HFFs were infected with HCMV at an MOI of 1 and examined by electron microscopy at 7 days postinfection (dpi). Briefly, the cells were fixed with 2.5% glutaraldehyde and postfixed with 1% osmium tetroxide. Cells were then stained with 2% uranyl acetate, dehydrated with a graded series of acetone, infiltrated, and embedded in Epon. Seventy nanometer sections were stained with lead citrate and examined using a CM10 electron microscope (Philips).

### Lipidomics.

For lipidomic analysis, lipids were extracted from WT and IFI16 KO HFFs under fully confluent serum-free medium and infected or not with HCMV for 96 h (MOI of 1 or 3) using an acidified chloroform-methanol-water mixture and glass beads. Briefly, 600 μL of chloroform-methanol (1:2 [vol/vol]) and 10 μL of formic acid and glass beads were added to the cells, vortexed for 20 s, and shaken for 5 min (2,000 rpm, 4°C). Next, 200 μL of chloroform and 350 μL of deionized water were added and shaken for an additional 20 min (2,000 rpm at 4°C). Subsequently, extracts were centrifuged (10 min, 3,900 × *g*), and the lower organic phase was collected and transferred to a chromatographic vial with a glass insert. In parallel with the biological samples, extraction was carried out with samples without cells to obtain extraction blanks to be used as negative controls.

After extraction, cells were analyzed using an Agilent 1290 LC system equipped with a binary pump, online degasser, autosampler, and a thermostated column compartment coupled to a 6540 Q-TOF–MS with a dual electrospray ionization (ESI) source (Agilent Technologies). Lipids were separated using reversed-phase column (Poroshell 120 EC-C8, 2.1 by 150 mm, 1.9-μm particle size; Agilent InfinityLab; Agilent Technologies) with a 0.2-μm in-line filter. The column was maintained at 60°C. The mobile phase comprised component A (5 mM ammonium formate in water-methanol [20/80, vol/vol]) and component B (2-propanol). The mobile phase was pumped at a flow rate of 0.3 mL/min. The gradient elution program was initiated with 20% of component B, which was ramped to 30% from 0 to 20 min and then from 30% to 100% from 20 to 30 min and kept for 1 min at 100% B. The column was then equilibrated with the starting conditions for 10 min. The total run time was 41 min, and the injection volume was set to 1.5 μL. Each extract was injected in duplicate. The data were collected in the positive-ion mode using the SCAN acquisition mode in a range from 100 to 1,700 *m/z* in the high-resolution mode (4 GHz).

MS analysis was carried out using the following parameters: capillary voltage, 3,500 V; fragmentation voltage, 120 V; nebulizing gas, 35 psi; drying gas temperature, 300°C. MS/MS analysis was performed using identical chromatographic and ion source conditions. The collision energy was set to 35 V and 80 V. The two most abundant peaks were selected for fragmentation and excluded for the next 0.3 min. The MS/MS spectra were acquired in the *m/z* range of 50 to 1,700. Lipid extracts were injected randomly using one quality control (QC) sample (pooled extracts) injected every 4 real samples for the LC-MS stability control.

Lipidomic data were prepared in the Agilent MassHunter Workstation Profinder 10.0 (Agilent Technologies). The MFE algorithm was used to extract the total molecular features (MFs) from the raw LC-MS data using the following parameters: ion threshold, >1,000 counts; ion type, H^+^; isotope model, common organic (no halogens); charge state range, 1 to 2; MFE score, ≥70. Next, the list of identified lipids was used for the Targeted Molecular Feature Extraction step with the following parameters: positive ions, charge carriers, H+, Na^+^, NH_4_^+^; match tolerance, 20 ppm; retention time, 0.2 min; Gaussian smoothing before extracted ion chromatogram extraction (EIC) filtering on peak height, 1,000 counts. The .cef files were exported and imported to Mass Profiler Professional 15.1 software (Agilent Technologies) for data alignment and filtration. Missing values were exported as missing. The alignment parameters were set as follows: alignment slope, 0.0%; alignment intercept, 0.2 min; mass tolerance slope, 20.0 ppm; intercept, 2.0 mDa. Filtration was based on frequency—the MFs remained in the data set if they were present in 80% of the samples in at least one specified group—and on the QC %RSD—MFs remained if the %Relative Standard Deviation (RSD) was <25% in all the QC samples. The MFs present in the extraction blank with an average peak volume higher than 10% of the average peak volume in the real samples were removed.

The statistical analysis and fold change calculation were conducted using Mass Profiler Professional 15.1 software (Agilent Technologies) and MetaboAnalyst5.0 (https://www.metaboanalyst.ca/home.xhtml). The parameters in the statistical test (Mann-Whitney unpaired test or Student's *t* test were a *P* value of ≤0.05 and asymptotic *P* value computation, and only detectable values were used for the calculation of fold change and *P* value). Lipid identification was carried out using the two-step procedure: (i) a custom lipid database (containing the mass of theoretical lipid structures) search based on an accurately measured *m/z* value (Δ5 ppm tolerance) and (ii) manual interpretation of the obtained MS/MS spectra. The identified lipid species were described according to the lipid class, number of carbon atoms, and number of double bonds in fatty acyl substituents. The diagnostic ions for the lipid class confirmation were as follows: *m/z* 184.0726 for confirmation of the PC identity, neutral loss of 141.02 Da for the confirmation of the PE identity, neutral loss of 185.01 Da for the confirmation of PS identity, and *m/z* 369.3536 for confirmation of CE.

### Statistical analysis.

Statistical tests were performed using GraphPad Prism version 5.00 for Windows (GraphPad Software), Mass Profiler Professional 15.1 software (Agilent Technologies), or MetaboAnalyst5.0 (https://www.metaboanalyst.ca/home.xhtml). The data are presented as means and standard errors of the means (SEM). Differences were considered statistically significant when the *P* value was <0.05.
